# Barriers and Opportunities for Sustainable Hand Hygiene Interventions in Rural Liberian Hospitals

**DOI:** 10.3390/ijerph18168588

**Published:** 2021-08-14

**Authors:** Lucy K. Tantum, John R. Gilstad, Fatorma K. Bolay, Lily M. Horng, Alpha D. Simpson, Andrew G. Letizia, Ashley R. Styczynski, Stephen P. Luby, Ronan F. Arthur

**Affiliations:** 1 School of Medicine, Stanford University, Stanford, CA 94305, USA; ltantum@stanford.edu (L.K.T.); lily.horng@gmail.com (L.M.H.); astyczyn@stanford.edu (A.R.S.); sluby@stanford.edu (S.P.L.); 2 School of Medicine, Uniformed Services University of the Health Sciences, Bethesda, MD 20814, USA; john.gilstad@usuhs.edu; 3 National Public Health Institute of Liberia, Monrovia 1000, Liberia; director.libr@gmail.com; 4 Q&A, Inc., Monrovia 1000, Liberia; asimpson@qnaservices.com; 5 Naval Medical Research Center, Silver Spring, MD 29010, USA; andrew.g.letizia.mil@mail.mil

**Keywords:** hand hygiene, infection prevention and control, sustainability, hospital safety, mixed methods, Liberia

## Abstract

Hand hygiene is central to hospital infection control. During the 2014–2016 West Africa Ebola virus disease epidemic in Liberia, gaps in hand hygiene infrastructure and health worker training contributed to hospital-based Ebola transmission. Hand hygiene interventions were undertaken post-Ebola, but many improvements were not sustainable. This study characterizes barriers to, and facilitators of, hand hygiene in rural Liberian hospitals and evaluates readiness for sustainable, locally derived interventions to improve hand hygiene. Research enumerators collected data at all hospitals in Bong and Lofa counties, Liberia, in the period March–May 2020. Enumerators performed standardized spot checks of hand hygiene infrastructure and supplies, structured observations of hand hygiene behavior, and semi-structured key informant interviews for thematic analysis. During spot checks, hospital staff reported that handwashing container water was always available in 89% (*n* = 42) of hospital wards, piped running water in 23% (*n* = 11), and soap in 62% (*n* = 29). Enumerators observed 5% of wall-mounted hand sanitizer dispensers (*n* = 8) and 95% of pocket-size dispensers (*n* = 53) to be working. In interviews, hospital staff described willingness to purchase personal hand sanitizer dispensers when hospital-provided supplies were unavailable. Low-cost, sustainable interventions should address supply and infrastructure-related obstacles to hospital hand hygiene improvement.

## 1. Introduction

Hospital-acquired infection endangers the health of patients, healthcare workers, and community members [1,2]. The risk of hospital-acquired infection is particularly high in low- and middle-income country (LMIC) settings, with the prevalence of hospitalacquired infection estimated at 15.5 infections per 100 patients in LMICs compared to 4.5 infections per 100 patients in high-income countries [3]. Hand hygiene is a key tool for interrupting infection transmission in the health care environment [4]. However, health worker hand hygiene practices are often sub-optimal, especially in low-income settings: in one multi-site baseline assessment, health workers at LMIC sites performed hand hygiene during 22% of total opportunities on average, compared to 54% of opportunities at high-income country sites [5]. Studies in Ghana, Nigeria, Eritrea, and Bangladesh found that average health worker adherence to hand hygiene guidelines ranged from 1% to 44% using direct observation [6,7,8,9,10,11].

Poor adherence to hand hygiene guidelines may arise from a range of institutional- and individual-level factors including time constraints, lack of training, poorly aligned incentives, and the perception that hand hygiene is not important [12,13,14,15]. Healthcare workers also face barriers to hand hygiene when infrastructure and supplies are unavailable [16]. Globally, one-third of healthcare facilities do not have adequate hand hygiene infrastructure at the point of care; in the world’s 47 lowest-income countries, half of the facilities lack basic water services [17]. As a result, 1.8 billion people worldwide seek care in facilities without adequate access to water services [17]. A lack of hospital hygiene infrastructure may lead to worse outcomes for patients. At a district hospital in Rwanda, running water outages of one day or longer were associated with higher odds of surgical site infection among patients who underwent caesarean section [18].

In Liberian healthcare settings, basic handwashing materials such as running water, soap, and chlorine are often in short supply, raising the risk of hospital-based disease transmission [19,20,21]. Gaps in Liberia’s hospital hand hygiene infrastructure became apparent during the 2014–2016 West Africa Ebola Virus Disease (Ebola) epidemic. Many Liberian hospitals were ill-prepared to manage Ebola patients as they lacked sufficient personal protective equipment and handwashing material [22,23,24]. The Ebola epidemic had an outsized impact on Liberia’s health workforce: Ebola infection risk was up to 30 times higher among healthcare workers compared to the general population [25].

Interventions were undertaken in Liberian hospitals during and post-Ebola, such as the introduction of chlorine solutions for hand hygiene during outbreaks [26]. However, hospitals continued to face hand hygiene supply shortages, undermining epidemic preparedness [27]. The COVID-19 pandemic has highlighted the risks of poor hospital hygiene during public health emergencies [28]. Just as disproportionate healthcare worker infection occurred during the Ebola epidemic, healthcare workers in the COVID-19 pandemic comprised 16% of Liberia’s total confirmed COVID-19 cases throughout August 2020 [29]. In August and September 2020, according to local reports, nurses in multiple counties across Liberia held a strike to protest inconsistent salary pay and lack of personal protective equipment and safety protocols at their facilities amidst the threat of COVID-19.

Improvements in hospital hand hygiene in Liberia could protect health workers and patients from infection. Focused hospital-based interventions can improve hand hygiene practices and reduce the short-term risk of infection [5,30,31]. However, the long-term sustainability of interventions is difficult to attain in low-income settings, especially in rural low-income areas where access to essential infrastructure, supplies, and maintenance services is low [32,33]. In a post-Ebola hand hygiene training and infrastructure intervention in Liberia, median health facility infection control compliance scores increased by 6%, from 76% to 82%, during the three-year intervention period [34]. Weak supply chains, coupled with a lack of financing, threaten the longevity of hand hygiene intervention strategies in Liberian hospital settings [35]. Although some studies quantitatively assessed Liberia’s post-Ebola progress in implementing hospital infection control and training health workers [36,37], to date, barriers to and facilitators of sustainable hospital hand hygiene interventions have not been studied in-depth in Liberia.

We employed a mixed-methods approach to characterize post-Ebola hand hygiene infrastructure, supplies, and practices at seven health facilities in two counties in rural Liberia, early in the COVID-19 global pandemic. This study was the first phase of a larger planned project to evaluate and improve hand hygiene at hospitals in Liberia by designing and piloting interventions in consultation with local stakeholders. The objectives of this baseline evaluation were to (1) describe hand hygiene facilities and behavior at hospitals in rural Liberia; (2) characterize barriers to hand hygiene practices in Liberian healthcare settings; (3) evaluate opportunities and hospital readiness for interventions to improve hand hygiene.

## 2. Methods

### 2.1. Study Site and Population

In the period March–May 2020, researchers conducted a baseline evaluation of hand hygiene infrastructure, supplies, and behavior at all hospitals in Bong County and Lofa County, Liberia, a total of seven facilities. Hospitals were defined as health facilities with inpatient services. No facilities meeting the inclusion criteria declined to participate in the study. Study participants included hospital administrators, medical staff, nonmedical staff, and caregivers. Interview participants were purposively selected by category of role in the hospital to acquire representation from administrative and medical leadership, medical staff, logistical staff, and support staff.

### 2.2. Study Design

Data collection consisted of spot checks, structured observations, and in-depth interviews with hospital staff. This mixed-methods approach allowed for a standardized characterization of hand hygiene infrastructure and supplies across all study sites, as well as a qualitative exploration of barriers to and facilitators of hand hygiene at each facility. In the spot checks, a team of four Liberian research enumerators assessed baseline availability of hand hygiene materials and infrastructure at hospitals. Enumerators were experienced in quantitative and qualitative data collection and received training in study data collection instruments. During structured observations, enumerators assessed hand hygiene behavior at hospital entry/exit points. Direct structured observation of hand hygiene actions is an established method of measuring hand hygiene behavior [38,39]. To strengthen the consistency of structured observation data, enumerators conducted practice observations, during which they observed the same scenario, compared the data they collected, and standardized reporting.

Experienced qualitative research professionals conducted in-depth interviews to elicit hospital staff and caregiver perspectives on hand hygiene. Researchers created interview guides informed by the Integrated Behavioral Model for Water, Sanitation, and Hygiene (IBM-WASH) to develop and evaluate hygiene behavior change interventions [40]. IBM-WASH provides a framework for systematically examining three dimensions (contextual, psychological, and technology factors), which act on five levels (structural, community, household, individual, and habitual) to influence behavior. This framework has guided the development of hand hygiene interventions in other settings [41,42]. Researchers adapted IBM-WASH dimensions for use in hospital rather than community environments (Table A1).

### 2.3. Definitions

**Figure 1:**
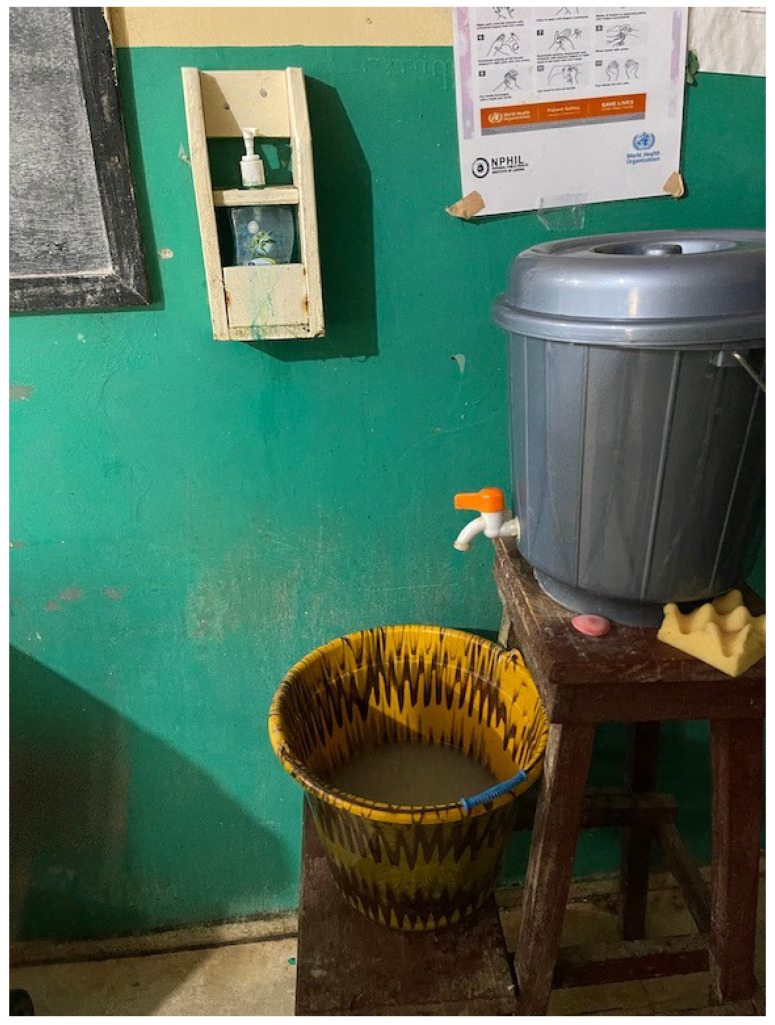
Handwashing station and wall-mounted hand sanitizer dispenser in Liberian health facility, 2020 (photo courtesy of the authors).

Handwashing stations were defined as water-filled containers with a closed top, a tap at the bottom, and a basin below to collect wastewater (Figure 1). Hand sanitizer dispensers were divided into three categories: wall-mounted dispensers, push-style dispensers not affixed to a wall, and pocket dispensers. Hand sanitizer dispensers were classified as “working” if they contained an adequate amount of hand sanitizer to perform hand hygiene and could operate as intended.

### 2.4. Data Collection

During spot checks, enumerators used a quantitative survey to record observational data on hand hygiene infrastructure and materials in hospital wards, hallways, bathrooms, and supply rooms; hospital water and power infrastructure; and hospital size. To collect information about typical hand hygiene material availability on wards, enumerators spoke with ward staff, inspected supplies and stores, and recorded whether staff described materials as being always, sometimes, or never available. Enumerators collected information about glove-wearing behavior among hospital staff by observing patient care interactions on all inpatient wards and recording whether staff wore gloves at pre-defined moments—before touching a patient, after touching a patient, and after touching patient surroundings—during each interaction.

Enumerators performed structured observations at each hospital entry/exit point where a handwashing container station was present. Using an Open Data Kit (ODK) instrument, enumerators recorded the type of handwashing material available at these stations. For each individual who passed by an entry/exit handwashing station, enumerators recorded whether the individual rinsed hands with water only, washed hands with soap, or did not perform hand hygiene. Observations took place over 2–4 days at each facility.

Researchers conducted qualitative in-depth interviews with consenting hospital administrators, health workers, and caregivers using semi-structured interview guides. Interviews took place within hospitals, were approximately 30–45 min in duration, and included questions related to individual hand hygiene practices and preferences, current and past institutional structures, and barriers to hand hygiene. Researchers also asked interview participants about their experience with hand hygiene practices during Ebola and COVID-19.

### 2.5. Data Analysis

Researchers generated descriptive statistics to characterize hand hygiene infrastructure, supplies, and practices at hospitals. Using spot check data, researchers calculated averages and ranges of the availability of hand hygiene materials and infrastructure at each hospital and overall. Researchers also used spot check data to calculate the proportion of patient care interactions during which hospital staff wore gloves.

Using data from structured observations, researchers assessed hand hygiene behavior at entry/exit handwashing stations. Since a range of hand hygiene materials was available at these stations, a handwash was defined as washing hands with bar or liquid soap or rinsing hands with water that was treated with detergent or chlorine. Researchers used z-tests with a significance level set at α = 0.05 to estimate differences in the proportion of individuals who washed hands at entry versus exit for each study hospital.

Interviews were audio-recorded, transcribed, and qualitatively analyzed for emergent themes. Researchers created a codebook using theory-driven codes derived from the IBM-WASH model and data-driven codes based on themes emerging from the initial review of interview transcripts [40,43,44]. To establish the reliability of the codebook, multiple researchers reviewed codes to identify differences in data interpretation and reach a consensus on coding protocol before coding each interview following codebook definitions [43]. When the initial interview coding was complete, researchers evaluated each code to systematically characterize prevailing viewpoints among interview participants and identify key quotes.

Researchers analyzed quantitative and qualitative data separately, then interpreted and triangulated the findings. During the triangulation process, researchers identified areas of agreement and dissonance between the findings that emerged from each method, allowing for the development of themes that encompassed findings from both methods [45].

### 2.6. Ethics

Data from interviews, structured observations, and spot checks were de-identified to prevent the identification of individual participants. Enumerators conducted structured observations of hand hygiene behavior in public settings and did not record individual identifying information. ODK-based data were uploaded directly to a secure server and were not saved on enumerators’ devices. Project methods were reviewed and approved by the National Research Ethics Board of Liberia (NREB), the Stanford University IRB, and the Uniformed Services University IRB.

## 3. Results

Researchers performed quantitative hand hygiene material spot checks, structured observations of hand hygiene at entry/exit points, and qualitative key informant interviews at seven hospitals in Bong and Lofa counties, Liberia. Data are presented in aggregate for all facilities to avoid the identification of specific facilities or staff members.

### 3.1. Spot Check Findings

During spot checks, enumerators observed a variety of materials and infrastructure to facilitate hand hygiene on hospital wards, in hallways, and in bathrooms (Table 1). All hospitals were powered by 1–2 working generators, and 5 also had access to solar power in part of the facility. One hospital had a generator that ran continuously. At other facilities, generators ran between 2 and 8 h a day, between 3 and 7 days a week.

Handwashing container stations contained a variety of hand hygiene materials, including bar soap, liquid soap, chlorinated water, and detergent-treated water. However, 8% of handwashing stations on wards or in hallways, and 70% of bathrooms, contained no water or handwashing materials. No station offered disposable towels. In addition to handwashing container stations, sink stations were available at five facilities, and hand sanitizer dispensers were available at all facilities. On average, eight pocket-size hand sanitizer dispensers—most of which were carried by individual staff members—were available at each facility. While 95% of pocket-size dispensers and 88% of push-bottle dispensers were working, only 5% of wall-mounted hand sanitizer dispensers were functional at the time of data collection (Table 1).

Handwashing container water and soap were reported to always be available at a majority of hospital wards, although piped running water was reportedly always available on only 23% of wards (Table 2). Few wards reported consistent availability of gloves (Table 2).

Hospital staff wore gloves during 91% (*n* = 308) of the moments observed during patient care. Enumerators observed staff washing gloves with water and bar soap during 46% (*n* = 142) of the observed moments and changing gloves during 11% (*n* = 35) of observed moments.

### 3.2. Structured Observation Findings

Researchers observed hand hygiene behavior at 2–8 hospital entry/exit points per hospital. At entry/exit points where handwashing was available, 36% of handwashing stations provided water that had been treated with detergent or chlorine. Of the stations with treated water, 79% also provided bar or liquid soap. Sixty-four percent of handwashing stations provided non-treated water and bar or liquid soap. At all hospitals, individuals were more likely to wash their hands while entering the facility than while exiting (56% washed hands at entry vs. 19% at exit) (Table 3).

### 3.3. Key Informant Interviews

Seventy-three individuals across the seven study hospitals participated in key informant interviews. At each study site, interview participants included hospital administrators, medical staff, non-medical staff, and caregivers. No individual approached for an interview declined to participate. Hand-hygiene-related themes emerging from the interviews fell into three categories: hand hygiene knowledge and practices; hospital structures for hand hygiene; and sustainability of hand hygiene interventions (Table 4).

#### 3.3.1. Hand Hygiene Knowledge and Practices

All interview participants said that they washed their hands on a regular basis. Most reported that they washed hands before and after attending to a patient, and many described practices that aligned with WHO Five Moments for Hand Hygiene guidelines [38]. Staff drew motivation for hand hygiene from a perceived risk of hospital-acquired infection. Fifty-six said they washed hands to protect themselves from infections that patients might transmit. Twenty-four said they washed hands to protect patients from infection risks in the hospital setting. Eighteen said they conceptualized handwashing as a means of preventing disease from spreading into the wider community.

Willingness to purchase one’s own hand hygiene materials was found to be high. Four participants said they purchased locally produced soap, typically made using ash, for hand hygiene when hospital-supplied materials were unavailable. Two said they addressed the unavailability of paper towels by carrying their own towel. Twelve said they purchased and carried pocket-size hand sanitizer dispensers for use when soap and water were unavailable, reporting that this was a common practice among medical staff. Many staff described hand sanitizer as “expensive for caregivers and patients” (Accountant), but did not personally view hand sanitizer as expensive. Several weighed the price of materials alongside the cost, monetary or otherwise, of an infection.

“[Hand sanitizer is] not expensive in the sense that if you ain’t use it and you get sick, you will spend more”

(Infection control focal person).

Participants preferred hand hygiene materials that they perceived to be effective, easy to use, and readily available. Participants most frequently expressed a preference for soap and water, with 57 naming soap as a preferred material. Forty-three expressed a preference for hand sanitizer, with some saying they preferred hand sanitizer “when your hands are not visibly soiled” (Nurse). Participants liked that hand sanitizer was portable, quick to use, and did not require paper towels to dry hands.

“If you wash your hands, you cannot wipe your hands on anything but you just got to wait until it gets dry… So for me I prefer using the alcohol rub”

(Nurse/supervisor).

Comparatively few (*n* = 12) said they preferred to use chlorine, detergent, or disinfectant solutions for handwashing. Some felt that chlorine solution production was overly complex.

Many participants said they never forgot to wash their hands at the hospital, describing handwashing as an ingrained habit. However, some said that they forgot to wash hands on occasion due to human error, when they were pressed for time, or when supplies were unavailable. No interview participant admitted to frequent or purposeful non-adherence to hand hygiene guidelines, but some said that they had observed other staff and hospital visitors who refused to perform hand hygiene.

During the Ebola epidemic, Bong and Lofa County hospitals saw many Ebola patients. Many interview participants had treated Ebola patients during the epidemic and witnessed high rates of health worker infection and mortality due to inadequate hand hygiene and poor infection control measures.

“Those [staff] that died from this facility it was because of gloves. They gave care with their bare hands and there was no hand hygiene practices here”

(Infection control focal person).

Data collection took place in the weeks before and after the onset of Liberia’s initial COVID-19 outbreak in March–May 2020. More than three-quarters of participants (*n* = 59) said they had heard of COVID-19 and expressed some knowledge of transmission methods, symptoms, and/or prevention measures. Many felt that COVID-19 was “hard to detect” (Nurse) and “even more deadly as compared to Ebola” (Lab technician).

#### 3.3.2. Hospital Structures for Hand Hygiene

Some staff reported that at least a basic level of hand hygiene materials was always available, but others said that, at their facilities, materials were totally unavailable for periods of “a day or two” (Midwife), “two, three weeks” (Medical staff member), or “months and months” (Midwife). Many said that bar soap was most commonly available at their facilities as it was inexpensive, while liquid soap and hand sanitizer were more expensive and, therefore, difficult to procure. Water and power availability were reportedly inconsistent. When running water was unavailable, hospitals relied on container water. Staff reported that fuel for generators was sometimes unavailable due to lack of financing.

Staff reported that hospital administrators and the medical director were responsible for the general planning and implementation of hand-hygiene-related initiatives. At all hospitals, a full-time infection prevention and control focal person was responsible for maintaining hand hygiene supplies and monitoring staff adherence to handwashing guidelines. Medical staff notified the infection control focal person when hand hygiene materials were out of stock on wards. The focal person collaborated with pharmacists and cleaners to restock hand hygiene supplies.

Staff reported that hospitals received funding from the Liberian government and international organizations for hand hygiene materials. However, government funding allotments were often delayed or insufficient for hospital hand hygiene needs, and half of the interview respondents (*n* = 37) named financial constraints as a barrier to hand hygiene. When funding was unavailable, hospitals were unable to restock hand hygiene supplies. Several hospitals participated in a performance-based financing scheme, wherein good health worker performance led to increased hospital funding [46]. As performance-based financing funding varied with each quarter, so did the amount of funding available to purchase hand hygiene materials.

“If that material finishes before that three months, to get materials it can be difficult for us”

(Cleaner).

Staff said that financing difficulties had a large impact on hospital procurement strategies. Hospitals usually procured materials from larger vendors in Monrovia, as local shops could not accommodate large orders and payment delays. In addition, hospitals sporadically received hand hygiene materials directly from the government or international organizations.

Staff produced handwashing solutions by mixing water with laundry detergent, chlorine, disinfectant, or liquid soap. Several were knowledgeable of production methods for 0.05% chlorine solutions but said that their hospitals used these solutions infrequently or not at all.

#### 3.3.3. Sustainability of Hand Hygiene Interventions

According to staff, hand hygiene infrastructure and supply availability improved post-Ebola, often as a result of Liberian government and international organization interventions to support hospital infection control. While some improvements had been sustained, others deteriorated over time.

“Alcohol based hand rub is also important but in our setting now, it is hard to find. Now we have the holders all over in our wards as a project that we did that time, but no you will [not] find anything inside”

(Infection control focal person).

Many staff said that their facilities had installed water pumps in recent years, some with support from outside organizations. However, pumps faced maintenance issues and were often non-functional. Staff across four hospitals reported that piped running water systems were broken or non-functional at the time of interview.

“The only problem that we have had, and it was solved and now we face it again, is water”

(Medical director).

Staff at one hospital were knowledgeable of alcohol-based hand sanitizer production methods owing to a past training partnership with a Japanese hospital. In recent years, they had produced hand sanitizer, which they used at their own facility and sold to other facilities in the county. However, the facility was not producing hand sanitizer at the time of interview, as staff said the raw materials needed for production—particularly ethanol and chemicals needed to test the quality of the product—were difficult to procure.

The Liberian government and international organizations had conducted several handhygiene- and infection-control-focused training programs at hospitals, many in response to or in the aftermath of the Ebola epidemic of 2014–2016. The majority of the staff interviewed (*n* = 50) said they had participated in one or more of these trainings. Many said they still practiced the skills learned in training; a few felt that the techniques that they had been taught were overly tedious.

“Especially if for me I am caring for patients at the ER, to go and stand maybe go through all of those steps, maybe it may be a waste of time”

(Nurse).

Most staff said that the Ebola epidemic had brought a sense of awareness and urgency to handwashing. While some felt that post-Ebola handwashing improvements had persisted up to the present day, others said that practices fluctuated with time.

“After the Ebola, really the hand washing was little bit dropping, but when we started learning about this Coronavirus, then of course we got back on course”

(Laundry man).

## 4. Discussion

Findings from interviews, spot checks, and structured observations at Liberian health facilities suggest that hand hygiene practices are strengthened by a high level of knowledge and motivation for infection prevention among hospital staff, but undermined in both implementation and sustainability by funding shortages and infrastructure deficiencies. In interviews, staff expressed knowledge of WHO-recommended hand hygiene practices, with nearly all reporting that they had received training in hand hygiene. However, our mixed-methods approach revealed gaps in infrastructure availability and local supply chains, which compromise hospital hand hygiene at our study sites. In spot checks, bar soap was available at over half of handwashing stations on wards, but several stations had no handwashing materials. Piped running water was reportedly always available on just 23% of hospital wards. These findings make it clear that additional training will have little impact on hospital hand hygiene practices unless appropriate facilities are present and supply availability is sustained.

A 2017 evaluation of the international Ebola epidemic response found several lasting improvements in Liberia, including the establishment of an incident management system, the expansion of diagnostic laboratory capacity, and improved infectious disease surveillance [47]. These improvements to the public health system in Liberia have been crucial, but our findings indicate that interventions to strengthen basic hospital hand hygiene resources, supplies, and infrastructure have not been similarly long-lasting. In interviews, hospital staff reported that many hospital hand hygiene interventions put into place during Ebola had ceased when supplies ran out or infrastructure broke down.

Staff asserted that the greatest barrier to the sustainability of hand hygiene interventions was financial. The hospitals included in our evaluation were funded by a range of government and private sources, but all struggled with financing for hand hygiene, reflecting the lack of funding for public services and the fragile economic situation in Liberia [48,49]. Several study hospitals participated in a performance-based financing scheme through which they received financial incentives to meet performance targets on a quarterly basis. Performance-based financing was implemented in the majority of Liberian counties, starting in 2009 [46,50,51]. Quarterly variability in performance-based funding amounts may pose a barrier to hospital financial planning, inhibiting the sustainability of interventions from one quarter to the next.

In the absence of increased funding, staff mostly relied on inexpensive materials, such as laundry detergent and soap made from ash, for hand hygiene. Although low in cost and widely available, these solutions may adversely affect skin integrity with repeated use, potentially increasing risk of disease transmission [38]. Hand sanitizer is an alternative, approved material that can be produced with brief training and used in the absence of water infrastructure, providing a potentially viable alternative to improving hand hygiene practices in Liberian health facilities [30,35,52,53].

The placement of locally produced hand sanitizer on hospital wards has been associated with improved healthcare worker hand hygiene practices and decreased incidence of hospital-acquired infections in Uganda [54]. Given our finding that just 5% of wallmounted hand sanitizer dispensers contained hand sanitizer material, an intervention targeting hand sanitizer availability at the individual level, rather than the hospital level, may be met with more success. In interviews, many hospital staff cited hand sanitizer as a preferred hand hygiene material, and 12 reported purchasing pocket-size hand sanitizer dispensers with personal funds. During spot checks, we found that 95% of pocket-size dispensers (*n* = 53) were working, indicating that individuals are able and willing to maintain these materials, in contrast to wall-mounted dispensers. Interventions centered around the individual-level provision of hand sanitizer have been well-tolerated and accepted in the Ugandan community setting and Swiss healthcare setting [55,56]. Hand hygiene interventions which leverage staff motivation to utilize and maintain individual hand sanitizer dispensers could be feasible and effective in the Liberian hospital setting.

Additionally, the use of hand sanitizer may be an acceptable means of decontaminating gloves to allow for extended use in the setting of PPE shortages [57,58,59]. Decontaminating gloves is already commonplace among healthcare providers: in spot checks, we observed that healthcare workers who wore gloves performed hand hygiene on gloves during nearly half of observed moments during patient care. During COVID-19, the US Centers for Disease Control advised that latex and nitrile gloves could be treated with up to six applications of hand sanitizer to allow for extended use in crisis contexts [60]. Other strategies for glove disinfection, including the use of soap and water or dilute bleach, are less well substantiated [58,59,61].

### Limitations

This study is subject to limitations. During structured observations of hand hygiene behavior, individuals may alter their behavior due to the presence of the research team [62,63]. Adherence to hand hygiene practices and glove-wearing behavior recorded during the study may, therefore, be higher than adherence when individuals are not under observation. Furthermore, researchers did not establish inter-rater reliability for structured observations, although the consistency of observations was assessed during enumerator training and practice sessions. In interviews, social desirability may have led to reluctance among hospital staff to admit to non-adherence to hand hygiene practices or express opinions that differed from conventional hand hygiene guidance. This could lead to the overstatement of actual adherence to hand hygiene. The use of a pre-existing model (IBM-WASH) to structure interviews may have constrained the topics discussed in qualitative interviews; however, this model covered a broad range of topics and added dimensions to interview questions that may have otherwise been missed. Due to the timing of data collection, in March–May 2020, the COVID-19 pandemic may have contributed to increased awareness of the importance of handwashing, impacting the behavior that we observed at hospitals.

## 5. Conclusions

Weak institutional support for hospital hand hygiene undermines sustained healthcare worker and patient safety in rural Liberia. Hospitals face shortages of basic hand hygiene materials and struggle to maintain basic water and power infrastructure. Past hand hygiene interventions have lacked sustainability, hampering the formation of good hand hygiene habits among staff. Hand hygiene interventions should be multifaceted, seeking not just to enhance health worker knowledge but also support material availability, improve basic infrastructure, and promote behavioral habit formation. Interventions with potential for sustainability may be those which utilize low-cost solutions, do not rely on robust supply chains, and are informed by human-centered design. Local hand sanitizer production may be one such intervention. However, even a low-cost intervention may require initial financial investment, and long-term improvement to hand hygiene is difficult in fragile states where hospitals and other public institutions do not receive sufficient oversight and financial support. Healthcare worker capacity-building, as well as improvements to health financing structures, will be necessary to prevent future disease outbreaks and meet the 2030 target of universal access to basic WASH services across all healthcare facilities [17,46]. Future research should examine the feasibility of intervention strategies to improve hospital hand hygiene services and practices and, ultimately, reduce the incidence of hospital-acquired infection in Liberia and other low-income settings. 

## Figures and Tables

**Table 1:** Hand hygiene materials and infrastructure observed in 7 Liberian hospitals, 2020.

Hand Hygiene Materials and Infrastructure	Availability	
	Total Number across All Facilities (*n* = 7)	Facility Mean (Range) or %
**Total handwashing container stations**	105	15 (11–23)
**Wards with at least one handwashing container station**	45	94%
**Wards with at least one sink station**	12	25%
*Ward handwashing container stations include⋯*		
Water	95	91%
Disposable towels	0	0%
Chlorinated water	7	7%
Liquid soap	26	25%
Detergent-treated water	16	15%
Bar soap	60	58%
Alcohol hand sanitizer	0	0%
No handwashing material	8	8%
**Other hand hygiene infrastructure**		
Sink stations	14	2 (0–5)
Wall-mounted hand sanitizer dispensers	160	23 (0–69)
Wall-mounted dispensers working	8	5%
Hand sanitizer push bottles	25	4 (0–14)
Push bottles working	22	88%
Pocket-size hand sanitizers	56	8 (0–22)
Pocket-size hand sanitizers working	53	95%
**Total bathrooms**	56	10 (1–20)
*Hand hygiene in bathrooms includes⋯*		
Water for hand hygiene	14	25%
Disposable towels	0	0%
Chlorinated water	1	2%
Liquid soap	3	5%
Detergent-treated water	5	9%
Bar soap	13	23%
Alcohol hand sanitizer	0	0%
No handwashing material	39	70%

**Table 2:** Reported availability of hand hygiene supply on wards in 7 Liberian hospitals, 2020.

Supply Availability on Wards	Availability across Number	All Hospital Wards (*n* = 47) *%*
**Container water**		
Always	42	89%
Rarely	1	2%
Never	4	9%
**Piped running water**		
Always	11	23%
Rarely	4	9%
Never	32	68%
**Soap**		
Always	29	62%
Rarely	4	9%
Never	14	30%
**Drinking water**		
Always	22	47%
Rarely	2	4%
Never	23	49%
**Gloves**		
Always	15	32%
Rarely	12	26%
Never	20	43%

**Table 3:** Observed hand hygiene behavior at hospital entry and exit handwashing stations in 7 Liberian hospitals, 2020. Significance of differences tested using z-test.

Study Hospital	Individuals Entering Hospital and Washing Hands % (*n*)	Individuals Exiting Hospital and Washing Hands % (*n*)	Difference in Proportion Washing at Entry versus Exit (%)	*p* Value
Hospital 1	80 (127)	12 (52)	−68	<0.001
Hospital 2	65 (487)	49 (250)	−16	<0.001
Hospital 3	35 (265)	12 (227)	−23	<0.001
Hospital 4	61 (195)	30 (50)	−31	<0.001
Hospital 5	56 (249)	18 (158)	−38	<0.001
Hospital 6	65 (462)	4 (202)	−61	<0.001
Hospital 7	40 (377)	2 (188)	−38	<0.001

**Table 4:** Emergent themes and sample quotes from interviews with hospital staff in Liberia, 2020.

Hand Hygiene Knowledge and Practices	
*Emergent Theme*	*Sample Quote*
Knowledge and behavior	You rub the soap, you use your hands like this rub it in your palm, you use your thumb behind the hands with the fingers, the nails and what have you. - Laundry supervisor
Motivation for hand hygiene	After interacting with my patient⋯ if I don’t do hand hygiene, I will infect myself and I will take that infection and carry home to my family and my family will infect that entire community. - Ward supervisor
Hand hygiene material preferences	I like to use the soap and water when my hand is visibly dirty, and use the hand sanitizer when my hand is not visibly dirty. - Nurse
Self-reliance for materials	Hand sanitizer is the preferable method because you carry it everywhere with you; right now I’ve got some in my bag. - Infection control focal person
Ebola and Covid-19 practices	We should continue washing our hands so we cannot spread this disease all over. - Cleaner
**Hospital Structures for Hand Hygiene**	
*Emergent Theme*	*Sample Quote*
Supply availability	Hand sanitizers business is very slim. So we use soap and water. - Nurse
Power and water infrastructure	The water relies on electricity, so that the generator has to be on to pump water. So if you are having problem with the fuel and the generator is not running then the water will not be pumped. - Doctor
Staff roles for hand hygiene	What I do is to make sure the staffs are doing the right thing when it comes to patients’ care. - Infection control focal person
Financing and procurement	To get the money to get the materials, sometimes business office will say they don’t have money. - Infection control focal person
Production of materials	We got our hand washing bucket, we got our solution that they made with Tide [detergent] soap, Dettol [antiseptic liquid]. Sometime we place small chlorine in it. - Nurse
**Sustainability of Hand Hygiene Interventions**	
*Emergent Theme*	*Sample Quote*
Supply and infrastructure interventions	After the Ebola outbreak there were systems put into place like increasing the basin for hand washing on various wards. - Nurse
Training interventions	During Ebola time at the time the training was going around, they were able to teach us the various steps that you need to follow that every part of your hands will be touched. - Nurse
Behavior change over time	The handwashing is ongoing⋯ Ebola time maybe it was 100%, but now we can say it’s 80 to 75%. - Nurse
Barriers to sustainability	They placed hand sanitizers into various places on the wards⋯ It’s still there but it’s empty because of support. - Cleaner

## Data Availability

The data presented in this study are available on request from the corresponding author. The data are not publicly available due to privacy and ethical concerns related to identification of study sites or participants.

## Appendix A

**Table A1:** Integrated Behavioral Model for Water, Sanitation, and Hygiene (IBM-WASH) [40] adapted for Liberia hospital hand hygiene qualitative assessment, 2020.

Levels	Contextual Factors	Psychosocial Factors	Technology Factors
**Societal/structural**	Existing policy, guidelines, and regulations	Hospital leadership and advocacy	Logistics of acquiring soap, alcohol, and chlorine locally
**Community**	Access to water and hand hygiene resources	Work culture surrounding patient care and hand hygiene	Location and maintenance of water and hand hygiene infrastructure
**Interpersonal**	Roles/responsibilities within the hospital regarding infection control practices	Specific norms governing hand hygiene behavior	Sharing access to hand hygiene resources (e.g., dedicated sinks for staff but not patients?)
**Individual**	Education and training about hand hygiene	Motivations for hygiene including disgust or perceived threat of infections	Convenience or preferences for specific hand hygiene products
**Habitual**	Environmental supports and barriers to repeated hand hygiene (e.g., high patient load)	Existing habits and expectations (e.g., wearing gloves but touching multiple patients)	Ease and effectiveness of routine use of specific hand hygiene products (e.g., quick-drying alcohol rub)
